# C17 Prevents Inflammatory Arthritis and Associated Joint Destruction in Mice

**DOI:** 10.1371/journal.pone.0022256

**Published:** 2011-07-25

**Authors:** Connie Chao, Barbara Joyce-Shaikh, Jeff Grein, Mehrdad Moshrefi, Fahimeh Raoufi, Drake M. Laface, Terril K. McClanahan, Patricia A. Bourne, Robert H. Pierce, Daniel M. Gorman, Stefan Pflanz

**Affiliations:** 1 Department of Immunology, Merck Research Labs, Palo Alto, California, United States of America; 2 Department of Translational Research and Pharmacology, Merck Research Labs, Palo Alto, California, United States of America; 3 Department of Antibody Technology, Merck Research Labs, Palo Alto, California, United States of America; 4 Department of Bioanalytics, Merck Research Labs, Palo Alto, California, United States of America; 5 Department of Anatomic Pathology, Merck Research Labs, Palo Alto, California, United States of America; Agency for Science, Technology and Research - Singapore Immunology Network, Singapore

## Abstract

C17 was first described about ten years ago as a gene expressed in CD34+ cells. A more recent study has suggested a role for C17 in chondrogenesis and development of cartilage. However, based on sequence analysis, we believe that C17 has homology to IL-2 and hence we present the hypothesis that C17 is a cytokine possessing immune-regulatory properties. We provide evidence that C17 is a secreted protein preferentially expressed in chondrocytes, hence in cartilage-rich tissues. Systemic expression of C17 *in vivo* reduces disease in a collagen antibody-induced arthritis model in mice (CAIA). Joint protection is evident by delayed disease onset, minimal edema, bone protection and absence of diverse histological features of disease. Expression of genes typically associated with acute joint inflammation and erosion of cartilage or bone is blunted in the presence of C17. Consistent with the observed reduction in bone erosion, we demonstrate reduced levels of RANKL in the paws and sera of mice over-expressing C17. Administration of C17 at the peak of disease, however, had no effect on disease progression, indicating that C17's immune-regulatory activity must be most prominent prior to or at the onset of severe joint inflammation. Based on this data we propose C17 as a cytokine that s contributes to immune homeostasis systemically or in a tissue-specific manner in the joint.

## Introduction

C17 (Cytl1, C4orf4, Cytokine-like protein 1, Protein C17, UNQ1942/PRO4425) was first mentioned in the literature as a predicted secreted protein, the mRNA of which is expressed in human bone marrow- and cord blood-derived CD34+, but not CD34−, cells [1]. Also, C17 reportedly is one of several genes for which elevated mRNA expression was identified in pre-malignant prostate stromal cells [2]. Recently, C17 was shown to promote chondrocyte differentiation from murine mesenchymal stem cells [3]. Consistent with the idea of a role for C17 in chondrocyte biology, presence of C17 protein has been reported in human cartilage explants [4]. However, overall, available information about C17 is sparse.

Here, we present the hypothesis that C17 may be a previously unrecognized member of the interleukin-2 (IL-2) cytokine family and therefore may possess immune-regulatory properties. Based on our hypothesis and the notion of C17's role in cartilage formation and regeneration, we decided to employ the technology of hydrodynamic delivery [5], in order to test effects of C17 over-expression in the context of acute joint inflammation *in vivo*. Choice of the CAIA model of rheumatoid arthritis permitted us to focus primarily on the acute inflammatory phase of arthritic disease.

Our results demonstrate that C17 can significantly reduce acute joint inflammation as evidenced by reduction of disease incidence, clinical disease score, and diverse histological parameters. Furthermore, C17 blocks expression of several key inflammatory genes, as well as expression of genes associated with bone and cartilage destruction. Our results suggest that C17 contributes to maintenance of immune homeostasis and support the idea of considering complementation of existing arthritis therapies with C17.

## Materials and Methods

### Transient transfection and expression of C17 in 293 cells

Human or mouse C17 containing their respective native signal sequences was cloned into the pTT5 expression vector to yield a C-terminus dual tagged V5 peptide (GKPIPNPLLGLDST)/octa-histidine fusion (V5H8) expression construct. C17-V5H8 was then transfected into a 50 ml culture of 293Ebna cells at 1×10^6^ cells/ ml by mixing 50 ug plasmid DNA, 100 ug Polyethlenimine (PEI), 5 ml Opti-MEM Reduced Serum media and incubating for 15 min at RT. The PEI-DNA-cell mixture was shaken at 37 C, 120 rpm in a 5% CO2 atmosphere incubator in a polycarbonate 1 L Erlenmeyer Baffled Flask (Corning). Supernatant was collected 72 hrs post transfection and concentrated ten-fold with Amicon Ultra centrifugal Filters and buffer exchanged with PBS using same filters. Cell pellets were harvested and lysed using 5 ml of 1 M NaCl, 1 mM EDTA, 1% Triton and 10 mM Tris-HCl, brought up to 50 ml with PBS and concentrated in the same manner as the supernatant. The concentrated supernatant or lysate was analyzed by Coomassie (Simply Blue) and Western blot, followed by transfer onto nitrocellulose membrane. The blot was blocked in 2.5% non-fat milk (Carnation) and 0.05% Tween/PBS for 10 min. To detect C17-V5H8, a 1∶3,333 dilution of 6×His mAb-HRP Conjugate (Clontech #631210) was added to the blocking solution and the antibody was bound overnight at 4 C. The blot was washed with a solution of 0.05% Tween/PBS and was detected using Amersham ECL Western Blotting Detection Reagent (GE Healthcare; Pittsburgh, PA).

### Construction and production of GFP and C17-V5H8 minicircle vectors

GFP or full length murine C17-V5H8 was cloned into the p2øC31.RSV.hAAT.bpA plasmid, kindly provided by Dr. Zhi-Ying Chen (Stanford University, Stanford, CA). We modified the vector and introduced unique 5′ PmeI and 3′ PacI restriction sites to facilitate directional cloning of cDNA's. We used PCR amplification to place 5′ PmeI and 3′ PacI cloning sites on the cDNA's and ligate these with the modified minicircle producing vector. GFP or C17-V5H8 minicircle DNA was produced following the methods described by Chen et al. [5] with some minor modifications. For overnight cultures we inoculated 1 liter of Terrific broth containing 100 ug/ml Ampicillin and incubated 18 hrs shaking at 270 rpm. We followed the minicircle production method [6], precipitated cultures and stored at −80 C. We used endotoxin free Qiagen megaprep kits for DNA purification, with 120 ml volumes of solution P1, 2&3. Minicircle DNA was eluted from the column; isopropanol was added and stored at −20 C. DNA was precipitated by spinning at 12 K, 30′ at 4 C, rinsed with 70% ethanol, air dried and resuspended in 1 ml of endotoxin-free Tris EDTA. Minicircle DNA was dialized in Midi MWCO 3.5 kDa tubes overnight against Tris EDTA. Purified minicircle DNA was verified by restriction digestion and sequencing.

### Mice and *in vivo* hydrodynamic gene delivery

Male B10.RIII mice were purchased from The Jackson Laboratory and housed under sterile conditions at Merck Research Labs. Hydrodynamic injections were performed under approved IACUC protocol and as described [7]. Essentially mice were injected in the tail vein with 2–3 mls Ringer's solution (Baxter; Deerfield, IL) containing 20 ug of minicircle DNA. The total injection volume was equivalent to 10% of the mass of the animal. Injections were performed rapidly, within 5–7 seconds using a 3 ml syringe fitted with a 27-guage needle.

### Induction of arthritis

CAIA was induced in wild type 12–16 week old B10.RIII males 3–4 days after hydrodynamic injection with GFP or C17-V5H8 minicircle, as described above. The mice were administered an intravenous injection of 4-clone arthrogenic monoclonal antibodycocktail (Chondrex; Redmond, WA.) Animals were monitored and scored daily thereafter for swelling and redness in the paws using a 0–3 scoring system per paw (maximum score per animal is 12): 0 = normal; 1 = mild redness or swelling in one digit or joint; 2 = moderate swelling or redness in multiple digits or joints; 3 = severe swelling or redness in all digits and throughout the entire paw. The increase in thickness of the hind paw due to edema was measured using a mechanical caliper (Mitutoyo, USA).

### Histopathological assessment of arthritic paws

Hind paws were dissected at the hairline and fixed in cold 10% neutral buffered formalin for 2 days, followed by decalcification in 10% EDTA solution at 4 C for 7 days, then processed for paraffin embedding. 5 micron sections were stained with H&E and Safranin O. Histological scores for articular cartilage damage, cortical bone erosion (0–3 scale) or for reactive synovium, leukocyte infiltration, pannus formation, gestalt score (0–4 scale) were assigned as follows: 0 = normal, 1–3 or 1–4: increasing degree of severity, depending on the parameter). Scoring parameters were assigned by a pathologist who was blinded to the different treatment groups. Two randomly selected sections were scored per paw.

### Immunohistochemistry

IHC was performed using a standardized staining protocol on formalin-fixed, paraffin-embedded tissues using a monoclonal antibody against C17-V5H8.

### Micro-computed tomography (micro-CT) analysis

Formalin-fixed hind paws were briefly rinsed for 15 min with cold running water prior to scanning. microCT imaging was performed with GE eXplore Lotus micro-CT instrument. (GE Healthcare, Piscataway, New Jersey). Images were acquired at 27 µm isotropic voxel size with 720 projections by 360 degree scan, integration time of 2000 msec with three frames, photon energy of 80 KeV and current of 450 uA. Acquisition time was approximately 100 minutes per scan, followed by an hour of projection correction and volume reconstruction. Maximum intensity projection and 3D image rendering were generated through original volumetric reconstructed images using Microview software (GE Healthcare).

### Serum analyses

Whole blood, collected at the end of the experiment by cardiac puncture, was centrifuged in serum gel tubes (Sarstedt) for 15 min at 4 C. Serum levels of C17-V5H8 were determined by ELISA. Nunc MaxiSorp plates were coated overnight at 4 C with anti-V5 antibody (Invitrogen; Carlsbad, CA) in 50 mM sodium bicarbonate buffer, pH 9.6. Plates were washed 3 times with 0.05% Tween/PBS and blocked with 2% BSA, 0.5% Tween/PBS for 2 hrs at RT. Purified recombinant C17-V5H8 protein diluted in normal mouse serum (Jackson ImmunoResearch; West Grove, PA) was used as the standard. Serum from minicircle treated animals were diluted in 2% BSA, 0.05% Tween/PBS, and incubated for 2 hrs at RT. After washing, HRP anti-His (R&D Systems; Minneapolis, MN) was added for 1 hr. Plates were developed using TMB Peroxidase EIA Substrate (Biorad; Hercules, CA) and the color reaction was stopped with 1 M phosphoric acid. The OD at 450 nm was assessed on a SpectrMax 340PC microplate reader (Molecular Devices; Sunnyvale, CA) Serum levels of RANKL (R&D Systems,) and COMP (Immunodiagnostics ) were determined using commercially available kits.

### C17-V5H8 purification

Protein was purifed from cell culture supernatants using affinity chromatography beads against the poly-histidine tag.

### Gene expression analysis in paws

Frozen tissues were homogenized using a polytron homogenizer and processed for RNA isolation; qRT-PCR was performed as described previously [8] [9].

### Statistical analysis

The mean and SEM were determined using GraphPad Prism 4 software. Student's t-test and Tukey's test was used for statistical analyses.

## Results

**Figure 1:**
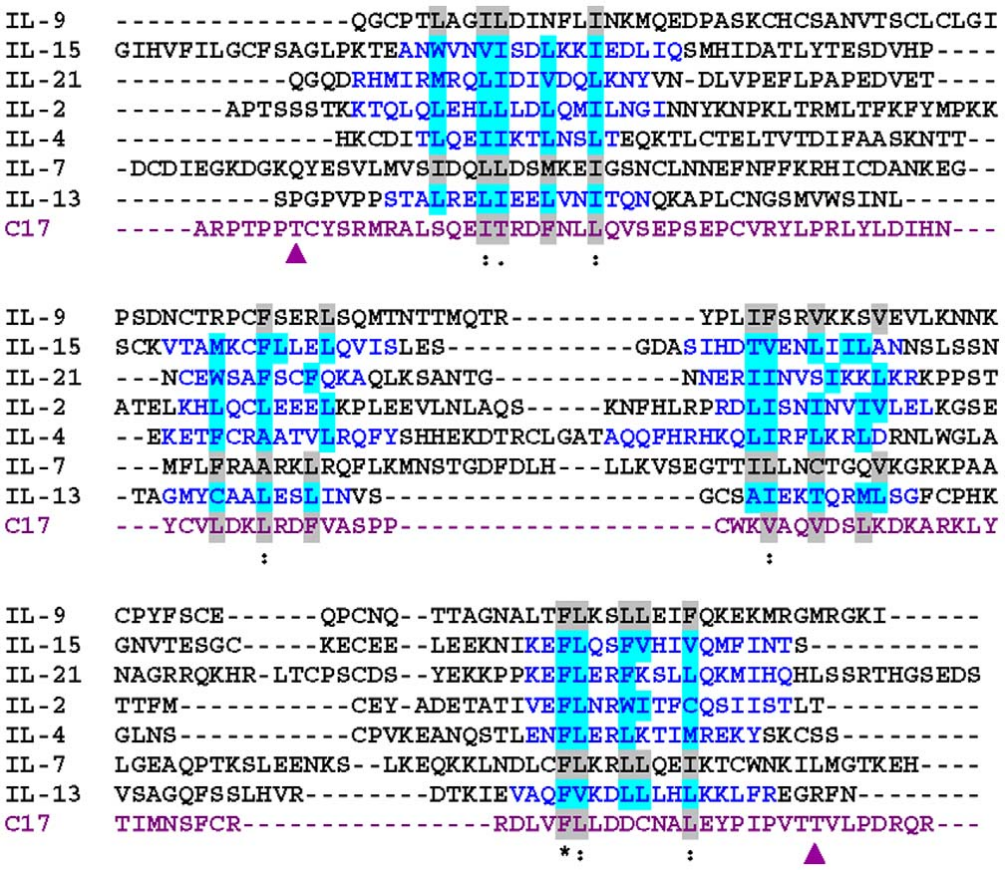
C17 is related to the family of common gamma chain-dependent cytokines. ClustalW multiple protein sequence alignment with some manual adjustment. Sequences of human cytokines, structurally related to IL-2, were aligned. C17 is highlighted in purple. Blue sequence denotes structure-confirmed alpha-helical secondary structure elements of respective cytokine (IL-2, PDB 2B5I; IL-4, PDB 1ITM; IL-13, PDB 1IKO; IL-15, PDB 2PSM; IL-21, PDB 2QQP). Light blue background confirms amino acid residues providing contacts to the respective protein core, according to afore listed structure data. Grey background denotes amino acid residues with predicted protein core contacts. Symbols *, :, . denote different levels of amino acid similarity/identity. The arrow heads indicate two residues for predicted O-linked glycosylation that are conserved between human and mouse C17.

C17 has first been mentioned in the scientific literature as a gene expressed in CD34+ cells about ten years ago [1]; to date, however, only quite limited information is available about its function. Systematic structure-guided protein sequence analysis of C17 reveals homology to the IL-2-related common gamma chain-dependent cytokines (Fig. 1). In addition, the genomic organization of exons and introns in the C17 gene locus, as well as the positioning of exon fusion sites relative to the predicted C17 secondary structure elements, is similar to the arrangement found for genes encoding other IL-2 cytokine family members. IL-2 and its close relatives have prominent roles in immune regulation [10] [11]. We therefore postulated that C17 may have the potential to modulate immune responses.

**Figure 2:**
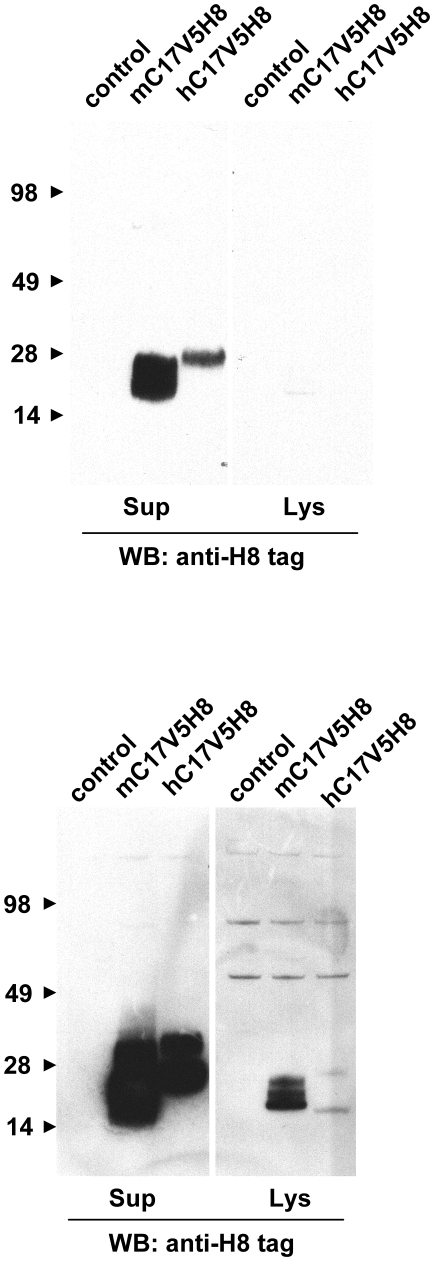
Human and mouse C17 are secreted proteins *in vitro*. HEK293 cells were transfected with eukaryotic expression vectors encoding C-terminally dual-tagged (V5H8 tags) human and mouse C17 protein. The tags were fused to the 3′ end of the full cDNA, retaining the natural N-terminal signal peptide and complete mature portion of C17. Supernatants and lysates were harvested and adjusted by volumes to permit direct comparison of protein quantities by Western blot. Proteins were separated by SDS-PAGE and visualized with an anti-poly His antibody. The same blot is shown twice with short (top) and long (bottom) exposure.

**Figure 3:**
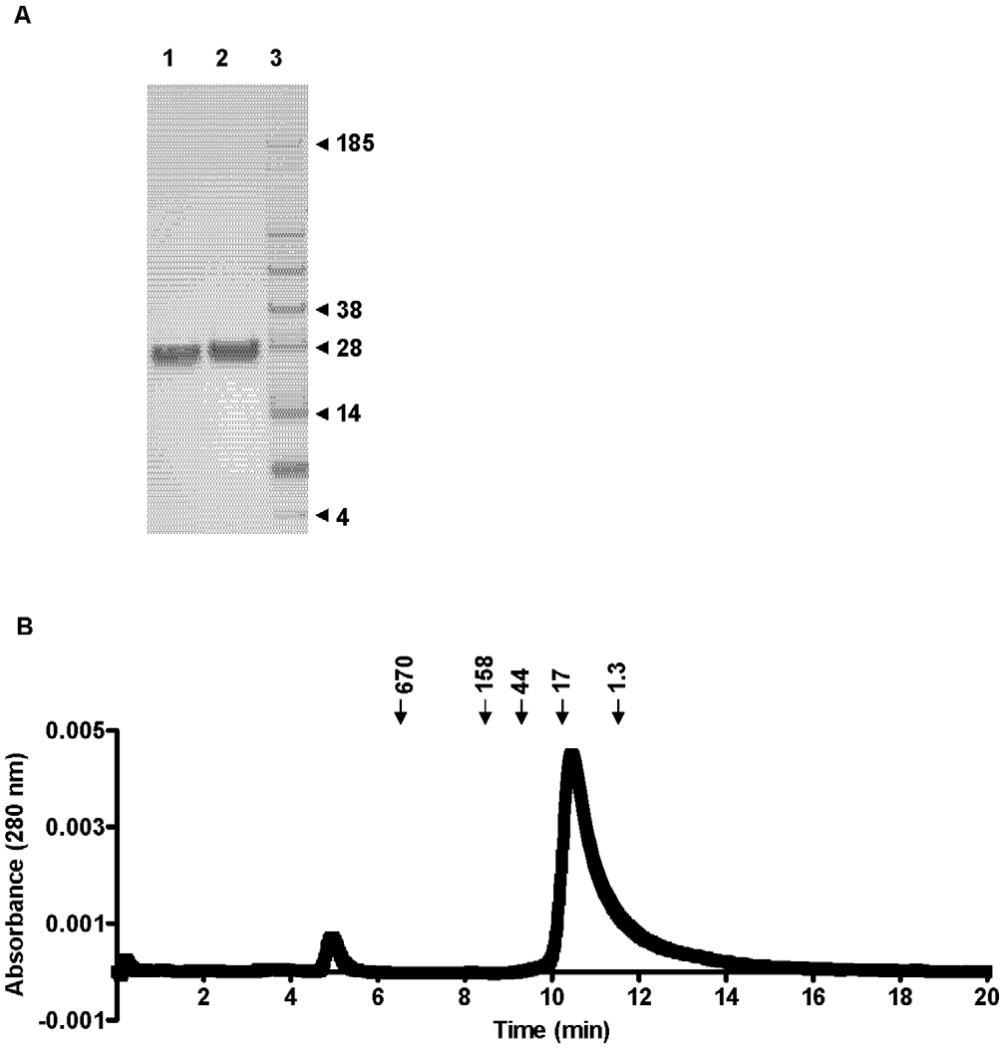
C17 protein is monomeric. (A) Recombinant purified human C17-V5H8 was analyzed by SDS-PAGE under reducing (1) and non-reducing (2) loading conditions and subsequent Coomassi staining. A protein marker is shown in the right column (3). (B) Baseline drift-corrected A280 nm elution profile of size exclusion chromatography after loading affinity-purified human C17-V5H8. Retention times of marker proteins (in kDa) are indicated above.

To verify the hypothesis that C17 is a secreted protein, we transfected HEK293T cells, a human embryonic kidney epithelial cell line that is frequently used for production of secreted proteins in the cellular supernatants after transient transfection, with a plasmid encoding mouse or human C17. We have not tried other cells or cell lines for expression of recombinant mouse or human C17 in our studies. Both constructs included the full length cDNA for C17 fused to two sequential C-terminal tags, V5H8. Western blot analysis of the cellular lysates and supernatants demonstrated C17 expression and secretion, respectively. The apparent mass of both mouse and human C17 in the supernatants is larger than expected (∼26 kDa vs 18 kDa) and is somewhat higher in the supernatants compared to the lysates suggesting that post-translational modifications of the C17 polypeptide may occur (Fig. 2). Consistent with these results, purified human C17-V5H8 has an apparent mass of approximately 26 kDa by SDS-PAGE (Fig. 3A). Biochemical characterization of affinity-purified human C17-V5H8 using size exclusion chromatography showed no evidence of dimers or oligomers, matching what would be expected for an IL-2-related cytokine (Fig. 3B).

**Figure 4:**
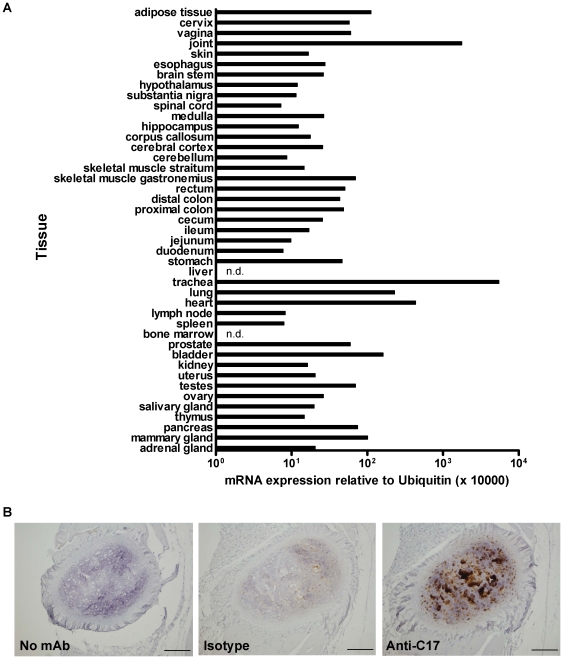
C17 mRNA and protein are expressed in cartilage-rich tissues and chondrocytes, respectively. (A) A variety of tissues were harvested and pooled from three C57BL/6 mice and then analyzed for C17 mRNA expression using qPCR (n.d., not detected). (B) Immunohistochemical staining for C17 on mouse sternal cartilage, using a mAb anti-C17, demonstrates protein expression by chondrocytes. Scale bars represent 0.1 mm.

In order to identify the most relevant sites of C17 expression *in vivo*, we profiled a wide array of mouse tissues. While C17 mRNA is detectable in various tissues, highest levels of mRNA were detected in cartilage-rich tissues such as trachea and joint (Fig. 4A).

We confirmed expression of C17 protein in chondrocytes using an anti-C17 monoclonal antibody (mAb) for immunohistochemical (IHC) staining (Fig. 4B). Our results expand on, and are consistent with, the reports of C17 mRNA and protein expression in cartilage [3] [4].

**Figure 5:**
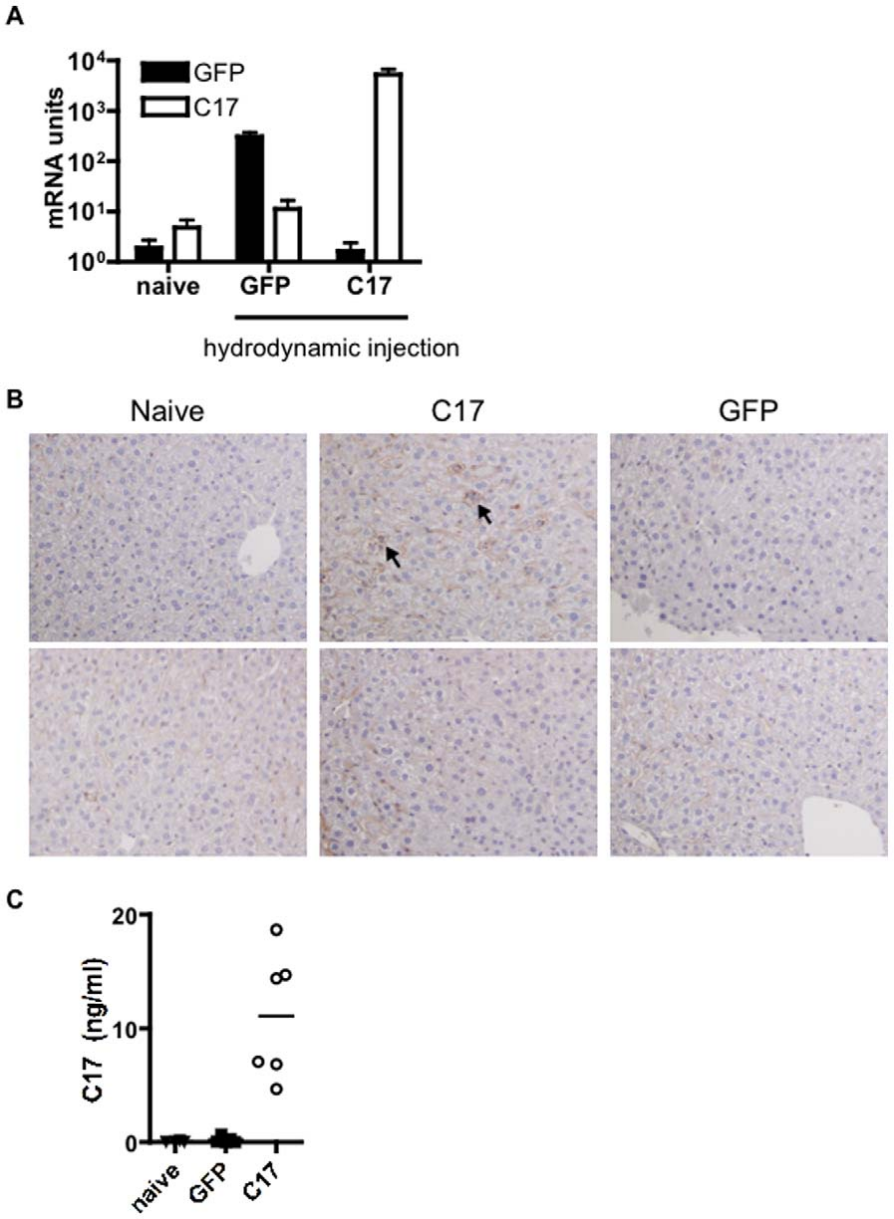
Sustained over-expression of C17 does not result in an inflammatory response *in vivo*. Age-and sex-matched mice received a hydrodynamic injection of C17 or GFP (control) minicircle, or were kept naïve. (A) After five weeks, animals were euthanized and hepatic over-expression of C17 mRNA was verified. In addition, (B) hepatic expression of C17 protein was confirmed by IHC using an anti-C17 mAb (top row) or isotype control Ab (bottom row); arrowheads highlight areas of positive C17 staining in C17-transfected, but not naïve or GFP-transfected liver; images were taken at 20× magnification. (C) Systemic exposure to C17 was verified by measuring serum levels of tagged C17 protein. Data shown represent a typical result from at least two independent experiments with five or more animals per group.

Next we sought to address the question if systemic over-expression of C17 *in vivo* would result in development of inflammation-associated pathology. For that purpose we applied the technology of hydrodynamic delivery of minicircle DNA. This method permits immediate and sustained hepatic transgene expression *in vivo* [5]. In lack of suitable antibody reagents for tracking of C17 protein in serum, we chose to use a minicircle encoding mouse C17 with the dual C-terminal tags, V5H8. With this tool, serum levels of the dual-tagged protein could be determined using a sandwich ELISA approach with mAbs recognizing the tags. Two control groups were used in this experiment: a group that was not injected, and a group that received minicircle encoding GFP. The mice were sacrificed after 35 days, and hepatic expression of C17 mRNA was verified after take down (Fig. 5A) Furthermore, IHC staining using an anti-C17 mAb confirmed hepatic C17 protein expression in C17-treated, but not GFP-treated mice. C17 staining was not observed in liver sections stained with an isotype control mAb (not shown). We also confirmed the presence of C17-V5H8 in the serum by ELISA (Fig. 5C). Necropsies were performed on the sacrificed animals and the following organs harvested: heart, aorta, lung, brain, thymus, liver, spleen, pancreas, kidney, stomach, small intestine, large intestine, cecum, tibia, sternum, trachea, cervical lymph node, skin. Tissues were embedded, sectioned and stained with H&E for histological analysis. No differences between the three groups were observed in systematic histological examination of sections generated from afore mentioned tissues (not shown). A panel of cytokines and chemokines was measured in the sera using a multiplex ELISA assay but no differences were observed (not shown). Mice of all groups gained weight in a comparable fashion during the five week experimental period (data not shown). Hence, no features suggesting a C17-dependent inflammatory response were observed in this experiment.

**Figure 6:**
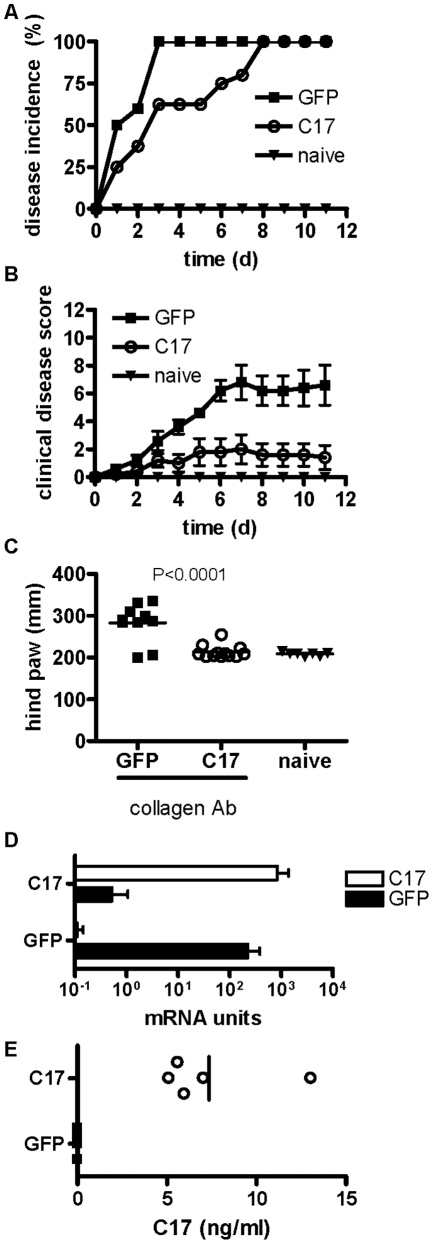
C17 over-expression protects mice from collagen antibody-induced arthritis. Age-matched male B10.RIII mice received a hydrodynamic injection of GFP or C17 minicircle DNA in Ringer's solution. Three days later mice received a single dose of the 4-clone arthrogenic mAb cocktail (3 mg) to induce CAIA (day 0), mice were followed for eleven days and then euthanized. (A) Incidence of disease and (B) mean clinical disease scores ± SEM are shown. Thickness of both hind footpads from each animal was measured with a mechanical caliper at the time of euthanasia (C). Quantitative RT-PCR was performed on liver tissues to verify hepatic expression of C17 mRNA (D), and protein levels in the serum were measured by ELISA (E). Data shown here are representative of at least three independent experiments with at least five animals per group.

As C17 is expressed in the joint and does not appear to act as a pro-inflammatory factor, we chose to investigate the impact of C17 expression in the context of CAIA, an *in vivo* model of acute joint inflammation and joint erosion. Three groups of mice were included in this study: one group received GFP minicircle on day −3 and arthrogenic cocktail on day 0, one group received C17-V5H8 minicircle on day −3 and arthrogenic cocktail on day 0, one group was kept naïve (ie, no minicircle or arthrogen). This timing was chosen because the murine liver appears to fully recover from hydrodynamic delivery within three days and no evidence of hepatic damage is detectable in the serum or by histology at this time point (data not shown). Typically, 100% disease incidence is reached at day 3–4 in this model, which was observed for the GFP group. Likewise, animals injected with Ringer's solution only developed disease after receiving the Ab cocktail to a similar extent and with 100% incidence (data not shown). In contrast, the C17 group had a delayed incidence with only 60% incidence on day 4 and 100% reached at day 8 (Fig. 6A). By day 7, the disease had reached its peak in the GFP treated mice, which demonstrated a mean clinical disease score of 6.8±1.2., whereas the mean score in C17-treated animals was 2±1.0. Clinical disease score was significantly reduced in C17-treated mice compared to GFP controls (p = 0.007), whereas the naïve mice had no clinical signs of disease, as expected (Fig. 6B). The degree of edema in the hind feet of mice was quantified on day eleven using a caliper. While C17 treated mice essentially showed no or only minimal swelling of the hind paws, GFP treated mice displayed significant edema (Fig. 6C). To confirm mRNA and protein expression for C17 during our study, samples of liver and serum were analyzed, respectively (Fig. 6D, 6E).

**Figure 7:**
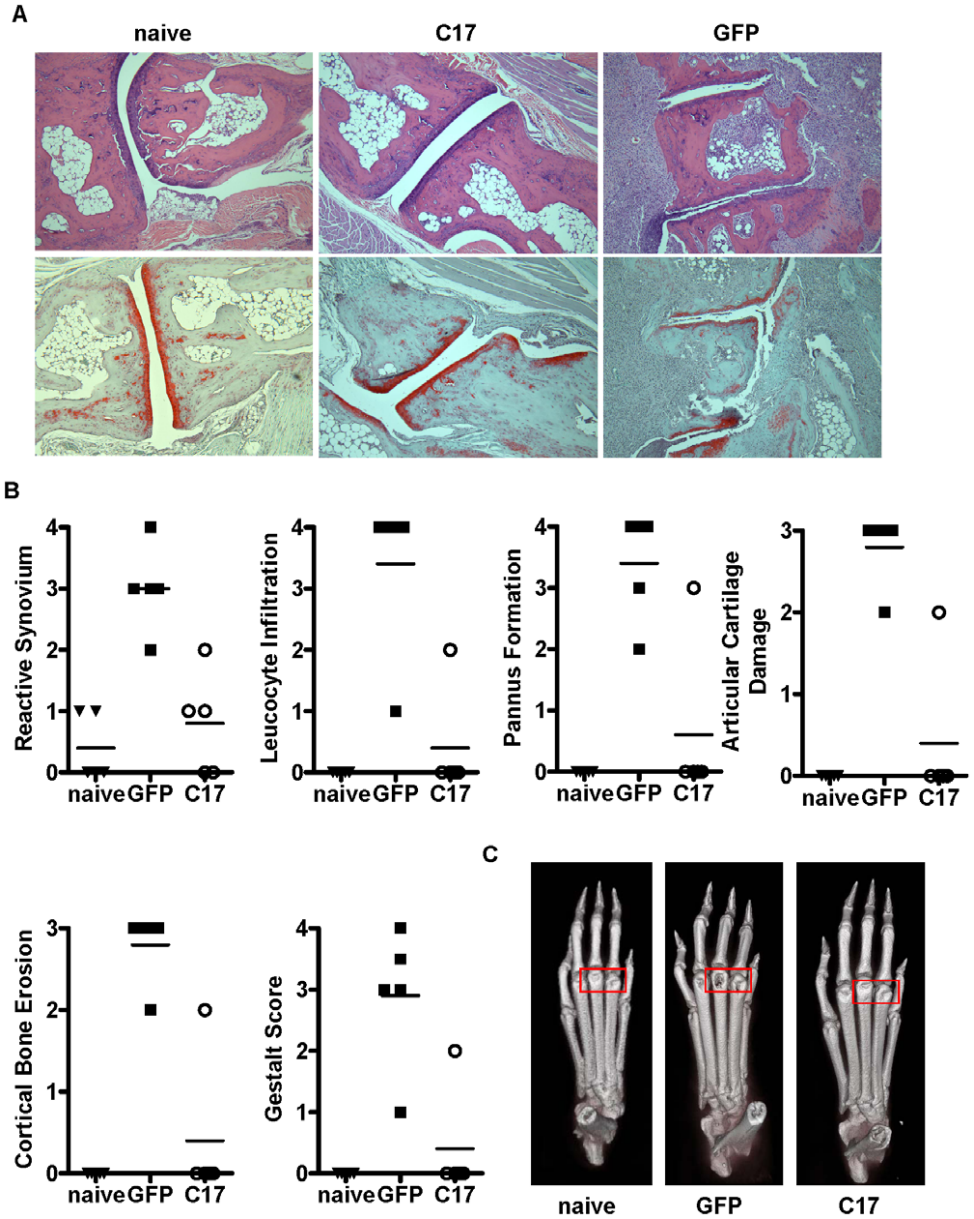
Bone erosion and histological parameters of arthritis are reduced in mice over-expressing C17. One hind paw per animal was harvested at the time of euthanasia and was analyzed by micro-CT and subsequent histology. (A) Representative histology images from individual hind paws are shown either stained with H&E (top row) or stained with Safranin O (bottom row). Images were taken at 10× magnification. (B) Scoring of disease severity according to diverse histological parameters. (C) Representive micro-CT scans are shown. Red box highlights prominent joint erosion in paw of GFP treated mouse. Similar data were generated in at least three independent experiments with at least five animals per group.

Upon sacrifice of the experimental animals, paws were embedded, sectioned and stained with H&E and then scored in a blinded fashion according to different histological criteria. Paws from GFP-treated animals showed features of severe arthritis, whereas paws from C17-treated animals were largely protected (Fig. 7A, 7B). Representative sections that were stained with H&E and with the cartilage-specific dye SafraninO underscore the protective effect of C17 on the joints (Fig. 7A). Paws were also harvested for micro-CT analysis. Paws from GFP control-treated mice showed considerable alterations in cortical bone density, with most apparent bone loss in the knuckle region. In contrast, paws from C17-treated animals were similar to naïve paws with minimal, if any, changes in bone structural integrity (Fig. 7C).

**Figure 8:**
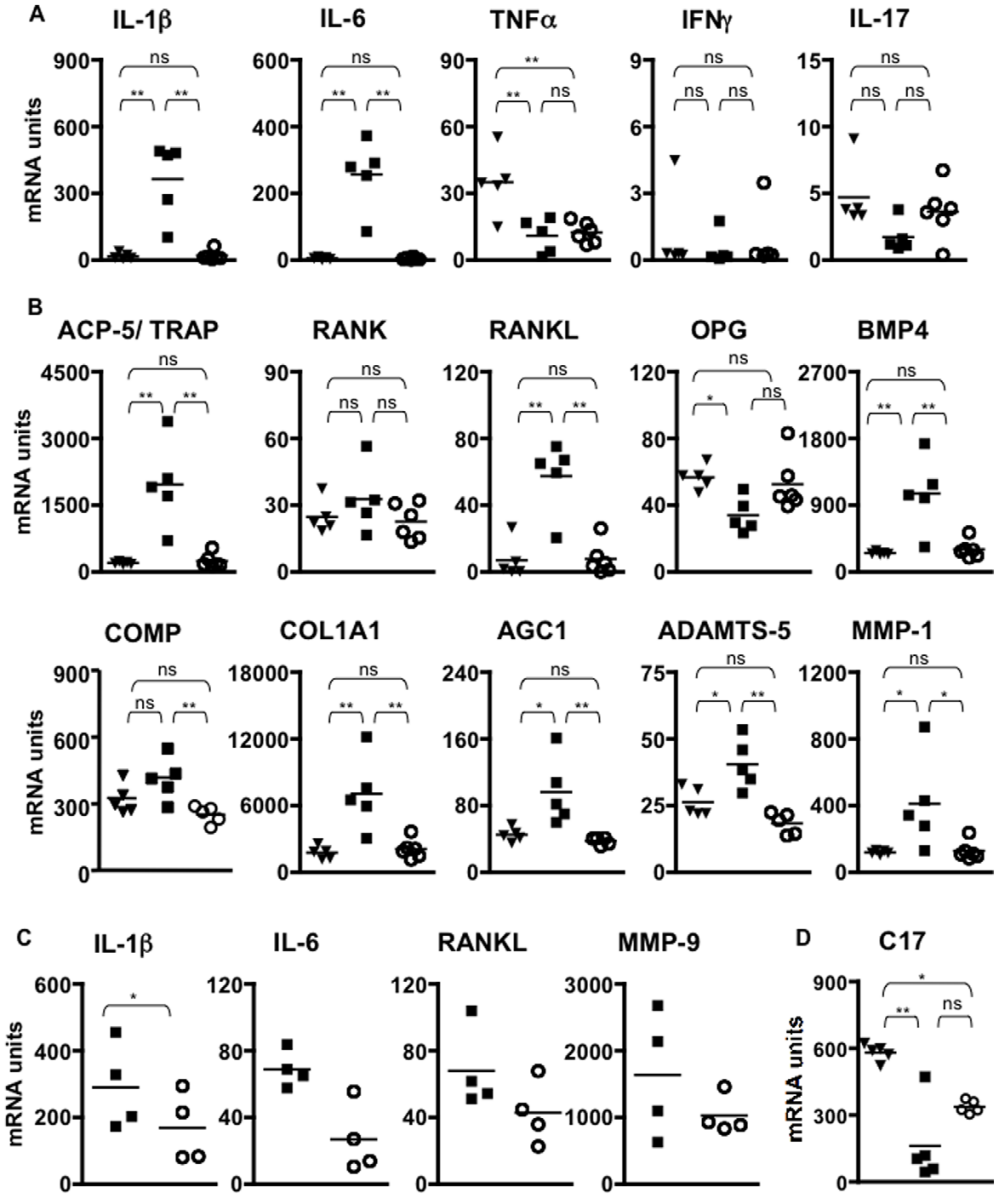
Reduced expression of inflammatory markers and genes associated with joint destruction in paws of C17-treated animals. One hind paw per animal was harvested at the time of euthanasia and quantitative RT-PCR was performed to measure mRNA levels of cytokines associated with joint inflammation (A), and genes associated with joint remodeling and arthritic tissue destruction (B). (C) mRNA expression of selected genes from paws with severe swelling and matched clinical disease score 3. (D) C17 expression in paws from naïve animals and animals that received arthrogen with GFP control or C17 minicircle. Values from naïve animals are shown as filled triangles, from GFP mice as filled squares, from C17 mice as open circles, throughout the figure. Not significant: ns; p<0.05: *; p<0.01: **. Similar data were obtained in at least two independent experiments with five or more mice per group.

As an additional level of analysis we performed gene expression profiling of the hind paws. We focused on some cytokine genes implicated with joint inflammation (Fig. 8A), and studied genes that are associated with pathologic tissue remodeling in the joint and/or bone (Fig. 8B). C17 dramatically reduced gene expression of IL-1b and IL-6, but had little impact on TNFa, IFNg and IL-17 (Fig. 8A). Generally, in the presence of C17, several other genes, typically associated with inflammation and pathological tissue remodeling in the joint, are kept to normal expression levels found in un-manipulated animals (Fig. 8B). This pattern of gene expression in the paw is consistent with the clinical disease scores and the results obtained from histological examination. Intriguingly, several inflammatory genes were trending to reduced expression with C17, even if paws matched for maximal clinical disease score were analyzed (Fig. 8C). C17 mRNA in the paw was reduced in mice receiving arthrogen compared to naïve mice, however, no significant difference occurred in GFP-treated compared to C17-treated animals (Fig. 8D).

**Figure 9:**
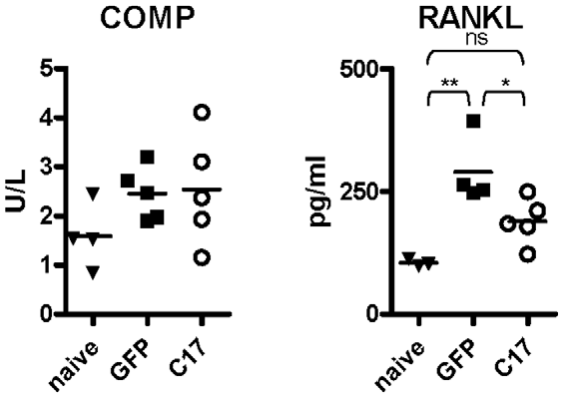
Serum levels of RANKL are reduced in C17 treated mice. At the time of euthanasia, serum was harvested from mice and analyzed for protein levels of the cartilage damage marker, COMP, and the marker of bone destruction, RANKL. Comparable results were obtained in at least two independent experiments with five or more mice per group. Only sera of mice that had analyte levels above the manufacturer's recommended sensitivity level of the respective assays are shown in the figure.

RANKL and cartilage oligomeric matrix protein (COMP) are serum biomarkers used for assessing bone and cartilage destruction, respectively. Serum levels of both markers were measured in the mice at the time of euthanasia: COMP serum levels were similar between naïve, and GFP and C17 treated groups receiving arthrogen. RANKL was elevated in the mice that had received the arthrogen compared to naive, and the concentration was lower in C17-treated compared to GFP-treated mice (Fig. 9).

**Figure 10:**
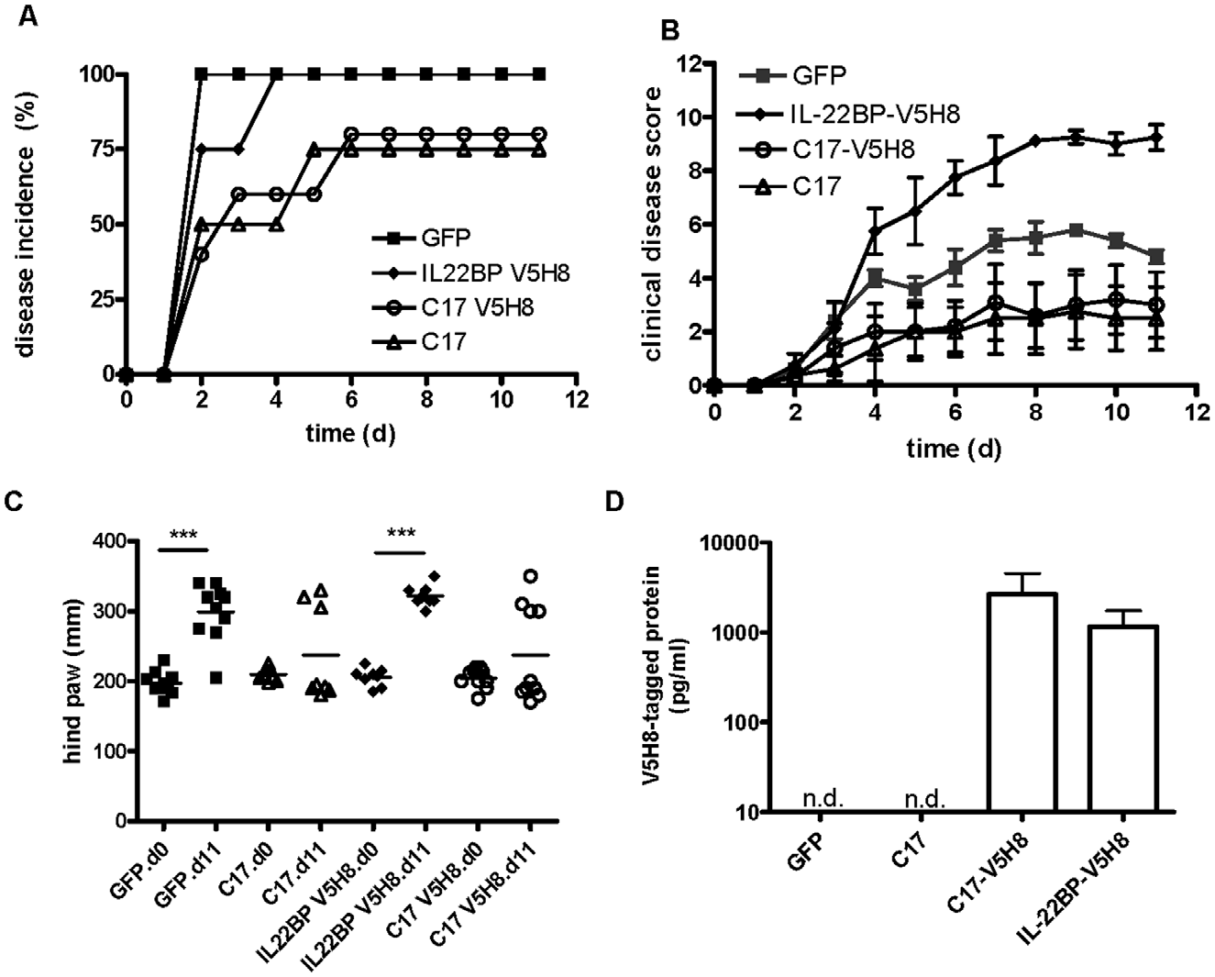
V5H8-peptide tag is not sufficient for protection from CAIA. Mice were injected with minicircle vector encoding GFP, V5H8-tagged IL-22BP, V5H8-tagged C17-V5H8,or untagged C17, on day −3. Artherogenic Ab cocktail was administered on day 0 and animals(n = 4–5/group) were monitored and scored daily relating to (A) disease incidence, and (B) clinical disease score. (C) Hind paw thickness was measured with a caliper on days 0 and 11, ***: p<0.001. (D) Presence of V5H8-tagged proteins in serum was verified on day 11; n.d., not detected.

To rule out the possibility that the V5H8-peptide tag attached to C17 was responsible for joint protection observed with C17, we performed an experiment in which, besides the C17-V5H8 and GFP, two control groups were included: untagged C17, and V5H8-tagged IL-22BP. As described above, minicircle vectors were administered at day −3, and the mice were challenged with the arthrogenic cocktail on day 0 and then followed for 11 days. Similar disease incidence and comparably mild joint swelling was observed in both groups, untagged C17 and C17-V5H8. GFP-treated mice developed maximal disease incidence and higher clinical scores than mice treated with either untagged C17 or C17-V5H8. However, mice receiving V5H8-tagged IL-22BP were most severely affected (Fig. 10). Therefore, this experiment provided no evidence for an anti-inflammatory effect caused by the V5H8 peptide tag.

**Figure 11:**
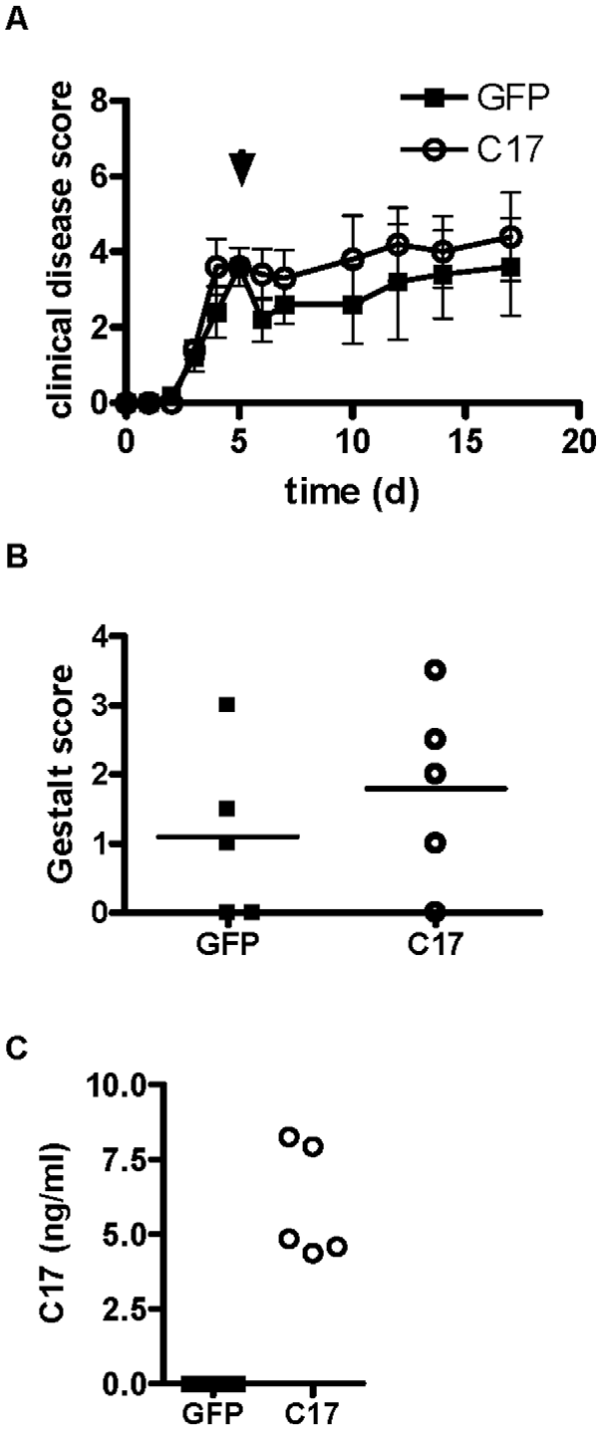
C17 over-expression does not modulate established arthritis. CAIA was induced in male B10.RIII mice with a single dose of 4-clone arthrogen on day 0, followed by hydrodynamic injection of GFP or C17-V5H8 minicircle on day 5 when mice had developed prominent paw inflammation. Mice were further monitored and euthanized at 17 days after initial disease induction. (A) The mean clinical disease scores including SEM are shown. (B) Histology gestalt scores were assigned to H&E-stained hind paws for GFP- and C17-treated animals. (C) Serum expression of C17 was verified. Data shown represent a typical result from at least two independent experiments with five or more animals per group.

The studies described above provide evidence for a potent anti-inflammatory and joint-protective effect of C17 if administered in a preventive setting. To investigate if C17 affects established joint inflammation we tested C17 over-expression in a therapeutic experiment. CAIA was induced on day 0 and minicircle vector encoding C17 or GFP were administered at day 5 when clinical disease symptoms are evident. Progression of clinical disease beyond day 5 was not different between GFP- and C17-treated groups (Fig. 11A). Paws were harvested from euthanized animals at day 17, and H&E stained sections were assessed according to disease severity by histology gestalt score of arthritic joint disease, but no differences between groups were observed (Fig. 11B). Systemic exposure to C17 was verified by confirming C17-V5H8 serum levels (Fig. 11C). Hence, this experiment did not provide evidence for a joint-protective effect of C17 when administered in the context of severe joint inflammation in established CAIA.

## Discussion

We decided to take a bioinformatics-based approach in an attempt to link C17 to a protein family and thus generate an initial working hypothesis. Two previous articles had predicted C17 as a secreted cytokine-like protein with alpha-helical secondary structure elements contained in the protein sequence [1] [3]. Several families of cytokines show structural features that involve alpha-helical bundle topologies, including IL-10-related cytokines, type I Interferons, common beta chain (CD131)-dependent cytokines, common gamma chain (CD132)-dependent cytokines, IL-6/12 related cytokines, and others [12] [13] [14]. Careful sequence analysis unveiled a close relationship of C17 to the IL-2-related, common gamma chain (CD132)-dependent, cytokines (Fig. 1).

Cytokines - by definition - are released from their respective producer cells as molecular messengers facilitating cell-cell communication. Hence, we set out to confirm that C17 is in fact a secreted molecule. Recombinant forms of both human and mouse C17 were detected in the supernatants after transfection of 293 cells. Of note, the secreted forms of both mouse and human C17 appear larger than the forms detected in the corresponding cell lysates (Fig. 2). This suggests post-translational modification of C17. The most common post-translational modifications introduced to secreted proteins are enzymatic glycosylations, either N- or O-linked, with the former being observed frequently among cytokine molecules. N-linked glycosylation can occur on asparagine residues which are part of the consensus motif NXS/T. O-linked glycosylation can occur on either serine or threonine residues; however due to a lack of a consensus motif, sites for O-linked glycosylation are difficult to predict. As protein sequences for both human and mouse C17 lack the consensus motif NXS/T, we propose that C17 undergoes O-linked glycosylation prior to its release. Interestingly, two threonine residues, one close to the N-terminus of the processed C17 and one close to its C-terminus, are conserved between human and mouse C17 protein sequences and are predicted sites for O-linked glycosylation (compare Fig. 1). Our results are in agreement with a report showing expression and secretion of mouse C17 in murine rib chondrocytes [3] (Fig. 4B), and of human C17 in transfected cells [1] (Fig. 2).

Biochemical analysis of affinity-purified C17 showed no evidence for formation of covalent or non-covalent oligomers (fig. 3A). Unlike e.g. IL-10, which requires formation of a homodimer for efficient receptor engagement, no evidence for oligomerization of common gamma chain (CD132)-dependent cytokines is reported in the literature. Likewise, our size exclusion chromatography data of purified C17V5H8 does not suggest formation of oligomers (Fig. 3B). Therefore, our findings are compatible with the hypothesis of C17 being an IL-2-related factor.

Based on this, we attempted to match C17 to various common gamma chain- related hematopoietic cytokine receptors employing biochemical methods, but no reproducible match could be identified (data not shown). Consequently, the cell surface receptor(s) for C17 are presently unknown and will be subject to future research.

We chose the method of hydrodynamic delivery of C17 minicircle in an effort to investigate the effects of C17 over-expression *in vivo*. Using this approach with minicircles encoding pro-inflammatory cytokines, e.g. IL-17, we have observed several key features of systemic inflammation including weight loss, increased blood leukocyte counts, tissue infiltration of activated lymphocytes and/or granulocytes, and increased systemic levels of inflammatory mediators (data not shown). However, even though *in vivo* over-expression of C17 for a five week period could be confirmed by measuring C17 the serum, none of the above features were recognized (Fig. 5). Of course, we have to formally consider the possibility that hepatocytes in this study may express a modified form of C17 and that therefore no overt effects were evident. In light of the data described in the CAIA model, however, such scenario appears unlikely.

Hence, we concluded that C17 does not act as pro-inflammatory mediator *in vivo*, but instead may possess immune-regulatory properties. An alternative interpretation of afore mentioned findings may be that C17 target cells require inflammation-associated signals to become sensitized to efficient C17 signaling. Therefore, C17 may have limited activity under homeostatic conditions. Owing this possibility, we selected a model of acute joint inflammation, CAIA, in which to test the effects of C17 over-expression.

Remarkably, expression of C17 resulted in delayed disease onset, minimized edema and arthritis histopathology score, bone protection, and reduced expression of many inflammation-associated markers (Fig. 6, Fig. 7, Fig. 8). In fact, according to most parameters, C17-treated mice that did receive the CAIA challenge were indistinguishable from age- and gender-matched naïve mice (Fig. 6, Fig. 7, Fig. 8). Considering the recent report that C17 supports chondrogenesis of mesenchymal stem cells [3], some level of C17-mediated cartilage protection in this model may be expected. However, our data suggest that C17 mediates joint protection on a broader scale, including suppression of inflammatory infiltrates, abrogation of pathological synovial reactivity, suppression of pannus formation, and protection of cortical bone. This indicates that C17 may elicit immune regulatory activity acting through cells other than chondrocytes and/or that C17 is capable of instructing chondrocytes to express a set of genes critical for control of local inflammation and joint homeostasis.

At the time of euthanasia, gene expression of a variety of markers associated with arthritic pathology was measured in the paws. Expression of IL-1b and IL-6 mRNA was significantly elevated in the paws of GFP control mice and reduced to background levels by C17 treatment (Fig. 8A). In contrast, TNFa was not different between GFP- and C17-treated animals. Unexpectedly, both groups receiving minicircle showed reduced TNFa mRNA relative to the naïve controls (Fig. 8A). These findings are in agreement with a previous study that reported sustained increase of IL-1b and IL-6 in this model; however, TNFa expression did not seem to be linked to disease progression [15]. IFNg and IL-17 levels were not different between groups (fig. 8A). As activated T cells are a dominant source for both IFNg and IL-17 and the CAIA model does not involve T cell priming, this result is perhaps to be expected.

Several markers of bone and cartilage remodeling were also analyzed. Expression of RANKL was increased in paws from GFP control mice, whereas in the presence of C17 protein, RANKL was reduced to levels observed in naïve animals (Fig. 8B). Receptor activator of nuclear factor kappa B (RANK) was not differentially expressed between groups and osteoprotegerin (OPG), the endogenous antagonist of RANK/RANKL interactions, was reduced in GFP-treated mice (Fig. 8B). As the RANK/RANKL pathway is critical to support maturation and activity of osteoclasts, this pattern of gene expression is consistent with the loss of cortical bone suffered by the GFP-treated animals (compare Fig. 7). In addition, expression of tartrate resistance acid phosphatase 5 (ACP-5/TRAP), a marker of osteoclast activity, was also significantly elevated in paws from GFP control animals but returned to base line in the presence of C17 (Fig. 8B). Osteoclast differentiation is supported by bone-morphogenic protein (BMP4) [16]. Our data shows significant up-regulation of BMP4 mRNA in paws from GFP-treated control mice, but not in C17-treated mice (Fig. 8B). Again, this finding supports the conclusion of minimized osteoclast activity in the presence of C17.

COMP, matrix-metalloproteinase 1 (MMP-1), a disintegrin and metalloproteinase with thrombospondin motifs 5 (ADAMTS5) are expressed by chondrocytes and are genes critically involved in cartilage remodeling and repair. As a marker for cartilage turnover, increases in COMP expression are indicative of increased cartilage remodeling. COMP expression is reduced in C17-treated animals (Fig. 8B). In mice, the aggrecanase ADAMTS5 is a potent mediator of cartilage destruction, as ADAMTS5 deficiency results in significant protection against cartilage erosion during experimental arthritis (Fig. 8B) [17] [18]. MMP-1 expression is also significantly elevated in GFP-treated mice but similar between naïve and C17-treated animals (Fig. 8B). MMP-1 expression by arthritic synovial fibroblasts can be triggered through IL-1b [19]. As we did observe elevated IL-1b levels, it is perhaps to be expected that MMP-1 expression is also elevated in the GFP group but not the C17 group.

Elevated expression of aggrecan (AGC1) and type I collagen (COL1A1), both of which are key components of articular cartilage, is indicative of cartilage remodeling. Again, consistent with increased cartilage turnover, expression of both genes is elevated in the GFP-treated but not C17-treated or naïve animals (Fig. 8B). Taken together, these gene expression profiles demonstrate increased articular cartilage remodeling and repair activity in this model, as expected, and, furthermore, support the notion that C17 can be protective against inflammation-associated cartilage breakdown in the joint.

When paws were matched for maximal clinical disease score (score 3), expression of arthritis-associated genes showed a consistent trend towards reduction in the presence of C17 with a significant reduction observed for IL-6 (Fig. 8C). This indicates that despite comparable clinical disease, C17 may still exert a joint-protective effect. C17 mRNA is reduced in joints of mice treated with arthrogen and the minicircle vector (Fig. 8D). Our data do not permit a conclusion of whether or not the observed reduction in C17 expression is a result of administration of the arthrogen and/or of the minicircle. However, GFP-treated mice trended towards reduced C17 mRNA, which may be explained by prominent cartilage destruction in those animals compared to C17-treated mice (compare to Fig. 7B).

However, C17 therapy had no effect on established CAIA. When administered on day 5 of the model clinical disease scores remained largely unchanged with GFP control, but also with C17 treatment (Fig. 11A). After animal sacrifice on day 17, gross histologic examination revealed no differences between groups: mice from both treatment groups showed inflammatory joint infiltrates, bone/cartilage destruction, and reactive synovia, resulting in similar histology gestalt scores (Fig. 11B).

These findings may be interpreted that C17 predominantly acts prior to, or at the very onset of, CAIA and increases the activation threshold for effector cells and/or alters their migration patterns. An alternative explanation could be that the robust and rapid inflammation triggered following delivery of the arthrogenic cocktail, may be too dominant to be overcome once disease is established.

In summary, our data demonstrate that C17 is sufficient for joint protection in a preventive setting but no protective effect was observed therapeutically. Whether perhaps C17 administration possesses therapeutic efficacy in milder or phenotypically different forms of experimental arthritis will have to be determined by future studies.
